# Diaqua­bis(4-oxo-1,4-dihydro­pyridine-3-sulfonato-κ^2^ *O* ^3^,*O* ^4^)zinc(II)

**DOI:** 10.1107/S1600536809044948

**Published:** 2009-10-31

**Authors:** Zhi-Biao Zhu, Shan Gao, Seik Weng Ng

**Affiliations:** a College of Chemistry and Materials Science, Heilongjiang University, Harbin 150080, People’s Republic of China; b Department of Chemistry, University of Malaya, 50603 Kuala Lumpur, Malaysia

## Abstract

In the crystal structure of the title compound, [Zn(C_5_H_4_NO_4_S)_2_(H_2_O)_2_], the 4-oxo-1,4-dihydro­pyridine-3-sulfonate anion chelates to water-coordinated zinc centres through the carbonyl O atom and through one O atom of the sulfonate group. The Zn^II^ atom lies on a center of inversion, and adjacent mol­ecules are linked by N—H⋯O and O—H⋯O hydrogen bonds, forming a three-dimensional network.

## Related literature

For the structure of the 4-oxo-1,4-dihydro­pyridine-3-sulfonate anion, see: Zhu *et al.* (2009 ▶).
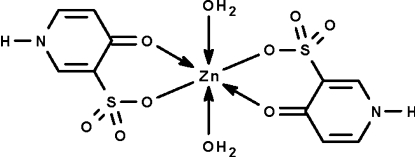


## Experimental

### 

#### Crystal data

[Zn(C_5_H_4_NO_4_S)_2_(H_2_O)_2_]
*M* *_r_* = 449.71
Monoclinic, 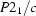

*a* = 4.9263 (1) Å
*b* = 20.9529 (6) Å
*c* = 7.4437 (2) Å
β = 98.9371 (9)°
*V* = 759.01 (3) Å^3^
*Z* = 2
Mo *K*α radiation
μ = 1.95 mm^−1^
*T* = 293 K
0.23 × 0.17 × 0.14 mm

#### Data collection

Rigaku R-AXIS RAPID IP diffractometer
Absorption correction: multi-scan (*ABSCOR*; Higashi, 1995 ▶) *T*_min_ = 0.662, *T*_max_ = 0.772
7334 measured reflections
1738 independent reflections
1629 reflections with *I* > 2σ(*I*)
*R*_int_ = 0.021

#### Refinement

*R*[*F* ^2^ > 2σ(*F* ^2^)] = 0.025
*wR*(*F* ^2^) = 0.074
*S* = 1.10
1738 reflections
127 parameters
3 restraints
H atoms treated by a mixture of independent and constrained refinement
Δρ_max_ = 0.45 e Å^−3^
Δρ_min_ = −0.46 e Å^−3^

### 

Data collection: *RAPID-AUTO* (Rigaku, 1998 ▶); cell refinement: *RAPID-AUTO*; data reduction: *CrystalClear* (Rigaku/MSC, 2002 ▶); program(s) used to solve structure: *SHELXS97* (Sheldrick, 2008 ▶); program(s) used to refine structure: *SHELXL97* (Sheldrick, 2008 ▶); molecular graphics: *X-SEED* (Barbour, 2001 ▶); software used to prepare material for publication: *publCIF* (Westrip, 2009 ▶).

## Supplementary Material

Crystal structure: contains datablocks global, I. DOI: 10.1107/S1600536809044948/bt5120sup1.cif

Structure factors: contains datablocks I. DOI: 10.1107/S1600536809044948/bt5120Isup2.hkl

Additional supplementary materials:  crystallographic information; 3D view; checkCIF report

## Figures and Tables

**Table 1:** Hydrogen-bond geometry (Å, °)

*D*—H⋯*A*	*D*—H	H⋯*A*	*D*⋯*A*	*D*—H⋯*A*
O1w—H1⋯O2^i^	0.84 (1)	2.05 (2)	2.797 (2)	147 (2)
O1w—H2⋯O4^ii^	0.84 (1)	1.92 (1)	2.744 (2)	171 (3)
N1—H3⋯O3^iii^	0.85 (1)	1.91 (1)	2.754 (2)	175 (3)

Symmetry codes: (i) 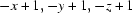; (ii) 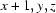; (iii) 
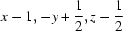
.

**Fig. 1.**
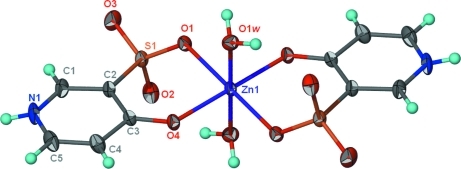
Anisotropic displacement ellipsoid plot (Barbour, 2001) of Zn(H2O)2(C5H4NO4S)2 at the 50% probability level; hydrogen atoms are drawn as spheres of arbitrary radius.

## supplementary crystallographic information

### Experimental

Zinc carbonate (0.25 g, 2 mmol) was added to a hot aqueous solution of 4-hydroxypyridine-3-sulfonic acid (0.35 g, 2 mmol); the pH value was adjusted to6 with 0.1 *M* sodium hydroxide. The solution was allowed to evaporate slowly at room temperature, and colorless prismatic crystals were isolated after about five days. CH&N elemental analysis. Calc. for C_10_H_12_N_2_O_10_S_2_Zn:*C* 26.71, H 2.69, N 6.23%; found: C 26.73, H 2.73, N 6.21%.

### Refinement

Carbon-bound H-atoms were placed in calculated positions (C—H 0.93 Å) and were included in the refinement in the riding model approximation, with *U*(H) set to 1.2*U*(C). The amino and water H-atoms were located in a difference Fourier map, and were refined isotropically with a distance restraint of N–H = O–H = 0.85±0.01 Å.

### Crystal data

**Table:**

[Zn(C_5_H_4_NO_4_S)_2_(H_2_O)_2_]	*F*(000) = 456
*M**_r_* = 449.71	*D*_x_ = 1.968 Mg m^−^^3^
Monoclinic, *P*2_1_/*c*	Mo *K*α radiation, λ = 0.71073 Å
Hall symbol: -P 2ybc	Cell parameters from 6865 reflections
*a* = 4.9263 (1) Å	θ = 3.4–27.5°
*b* = 20.9529 (6) Å	µ = 1.95 mm^−^^1^
*c* = 7.4437 (2) Å	*T* = 293 K
β = 98.9371 (9)°	Prism, colorless
*V* = 759.01 (3) Å^3^	0.23 × 0.17 × 0.14 mm
*Z* = 2	

### Data collection

**Table:**

Rigaku R-AXIS RAPID IP diffractometer	1738 independent reflections
Radiation source: fine-focus sealed tube	1629 reflections with *I* > 2σ(*I*)
graphite	*R*_int_ = 0.021
ω scans	θ_max_ = 27.5°, θ_min_ = 3.4°
Absorption correction: multi-scan (*ABSCOR*; Higashi, 1995)	*h* = −6→5
*T*_min_ = 0.662, *T*_max_ = 0.772	*k* = −27→27
7334 measured reflections	*l* = −9→9

### Refinement

**Table:**

Refinement on *F*^2^	Primary atom site location: structure-invariant direct methods
Least-squares matrix: full	Secondary atom site location: difference Fourier map
*R*[*F*^2^ > 2σ(*F*^2^)] = 0.025	Hydrogen site location: inferred from neighbouring sites
*wR*(*F*^2^) = 0.074	H atoms treated by a mixture of independent and constrained refinement
*S* = 1.10	*w* = 1/[σ^2^(*F*_o_^2^) + (0.0399*P*)^2^ + 0.5207*P*] where *P* = (*F*_o_^2^ + 2*F*_c_^2^)/3
1738 reflections	(Δ/σ)_max_ = 0.001
127 parameters	Δρ_max_ = 0.45 e Å^−^^3^
3 restraints	Δρ_min_ = −0.45 e Å^−^^3^

### Fractional atomic coordinates and isotropic or equivalent isotropic displacement parameters (Å^2^)

**Table:**

	*x*	*y*	*z*	*U*_iso_*/*U*_eq_	
Zn1	0.5000	0.5000	0.5000	0.01991 (11)	
S1	0.54548 (9)	0.35166 (2)	0.43742 (6)	0.02251 (13)	
O1	0.6943 (3)	0.41242 (6)	0.46588 (19)	0.0246 (3)	
O2	0.4026 (3)	0.33653 (7)	0.5890 (2)	0.0327 (3)	
O3	0.7182 (3)	0.30080 (7)	0.3867 (2)	0.0380 (4)	
O4	0.1877 (3)	0.47222 (6)	0.29786 (18)	0.0238 (3)	
O1W	0.7120 (3)	0.54012 (7)	0.3004 (2)	0.0279 (3)	
N1	−0.0061 (4)	0.30854 (10)	0.0136 (2)	0.0361 (5)	
C1	0.2078 (4)	0.30795 (10)	0.1490 (3)	0.0303 (4)	
H1A	0.3053	0.2703	0.1764	0.036*	
C2	0.2849 (4)	0.36189 (8)	0.2474 (3)	0.0221 (4)	
C3	0.1359 (4)	0.42039 (8)	0.2098 (2)	0.0209 (4)	
C4	−0.0876 (4)	0.41740 (10)	0.0613 (3)	0.0294 (4)	
H4	−0.1897	0.4540	0.0274	0.035*	
C5	−0.1522 (5)	0.36232 (11)	−0.0300 (3)	0.0353 (5)	
H5A	−0.2998	0.3615	−0.1245	0.042*	
H1	0.738 (5)	0.5794 (5)	0.318 (4)	0.035 (7)*	
H2	0.866 (3)	0.5233 (13)	0.301 (4)	0.040 (7)*	
H3	−0.081 (6)	0.2742 (9)	−0.028 (4)	0.051 (8)*	

### Atomic displacement parameters (Å^2^)

**Table:**

	*U*^11^	*U*^22^	*U*^33^	*U*^12^	*U*^13^	*U*^23^
Zn1	0.01975 (17)	0.01417 (16)	0.02420 (18)	−0.00106 (10)	−0.00165 (12)	−0.00169 (10)
S1	0.0258 (2)	0.0128 (2)	0.0270 (2)	0.00172 (16)	−0.00212 (18)	0.00056 (16)
O1	0.0221 (6)	0.0168 (6)	0.0333 (7)	0.0005 (5)	−0.0006 (5)	−0.0015 (5)
O2	0.0433 (8)	0.0247 (7)	0.0286 (7)	−0.0061 (6)	0.0009 (6)	0.0042 (6)
O3	0.0398 (8)	0.0203 (7)	0.0503 (10)	0.0124 (6)	−0.0037 (7)	−0.0046 (6)
O4	0.0226 (6)	0.0168 (6)	0.0295 (7)	0.0010 (5)	−0.0042 (5)	−0.0031 (5)
O1W	0.0263 (7)	0.0244 (7)	0.0333 (8)	0.0020 (6)	0.0059 (6)	0.0008 (6)
N1	0.0443 (11)	0.0297 (10)	0.0324 (10)	−0.0131 (8)	0.0006 (8)	−0.0141 (7)
C1	0.0366 (11)	0.0203 (9)	0.0337 (10)	−0.0038 (8)	0.0046 (8)	−0.0065 (8)
C2	0.0253 (9)	0.0178 (8)	0.0224 (9)	−0.0022 (7)	0.0008 (7)	−0.0024 (6)
C3	0.0215 (8)	0.0189 (8)	0.0212 (8)	−0.0032 (7)	−0.0005 (7)	0.0008 (6)
C4	0.0300 (10)	0.0295 (10)	0.0256 (9)	−0.0034 (8)	−0.0061 (8)	0.0025 (8)
C5	0.0364 (11)	0.0414 (12)	0.0248 (10)	−0.0108 (9)	−0.0056 (8)	−0.0042 (8)

### Geometric parameters (Å, °)

**Table:**

Zn1—O4	2.0618 (12)	O1W—H2	0.835 (10)
Zn1—O4^i^	2.0618 (12)	N1—C1	1.340 (3)
Zn1—O1^i^	2.1031 (13)	N1—C5	1.349 (3)
Zn1—O1	2.1031 (13)	N1—H3	0.846 (10)
Zn1—O1W^i^	2.1183 (15)	C1—C2	1.368 (3)
Zn1—O1W	2.1183 (15)	C1—H1A	0.9300
S1—O3	1.4496 (15)	C2—C3	1.434 (2)
S1—O2	1.4547 (17)	C3—C4	1.435 (2)
S1—O1	1.4681 (13)	C4—C5	1.352 (3)
S1—C2	1.7694 (19)	C4—H4	0.9300
O4—C3	1.274 (2)	C5—H5A	0.9300
O1W—H1	0.841 (10)		
			
O4—Zn1—O4^i^	180.00 (7)	C3—O4—Zn1	133.12 (12)
O4—Zn1—O1^i^	91.86 (5)	Zn1—O1W—H1	111.3 (18)
O4^i^—Zn1—O1^i^	88.14 (5)	Zn1—O1W—H2	112 (2)
O4—Zn1—O1	88.14 (5)	H1—O1W—H2	107 (3)
O4^i^—Zn1—O1	91.86 (5)	C1—N1—C5	121.09 (18)
O1^i^—Zn1—O1	180.0	C1—N1—H3	121 (2)
O4—Zn1—O1W^i^	90.38 (6)	C5—N1—H3	116 (2)
O4^i^—Zn1—O1W^i^	89.62 (6)	N1—C1—C2	121.0 (2)
O1^i^—Zn1—O1W^i^	88.76 (5)	N1—C1—H1A	119.5
O1—Zn1—O1W^i^	91.24 (5)	C2—C1—H1A	119.5
O4—Zn1—O1W	89.62 (6)	C1—C2—C3	120.70 (17)
O4^i^—Zn1—O1W	90.38 (6)	C1—C2—S1	115.75 (15)
O1^i^—Zn1—O1W	91.24 (5)	C3—C2—S1	123.06 (13)
O1—Zn1—O1W	88.76 (5)	O4—C3—C2	124.95 (16)
O1W^i^—Zn1—O1W	180.00 (7)	O4—C3—C4	120.14 (17)
O3—S1—O2	114.58 (10)	C2—C3—C4	114.91 (16)
O3—S1—O1	112.02 (9)	C5—C4—C3	121.1 (2)
O2—S1—O1	111.59 (9)	C5—C4—H4	119.4
O3—S1—C2	105.27 (9)	C3—C4—H4	119.4
O2—S1—C2	105.55 (9)	C4—C5—N1	121.12 (18)
O1—S1—C2	107.12 (8)	C4—C5—H5A	119.4
S1—O1—Zn1	123.21 (8)	N1—C5—H5A	119.4
			
O3—S1—O1—Zn1	170.03 (10)	O2—S1—C2—C1	−89.44 (17)
O2—S1—O1—Zn1	−59.98 (12)	O1—S1—C2—C1	151.53 (16)
C2—S1—O1—Zn1	55.08 (12)	O3—S1—C2—C3	−155.77 (16)
O4—Zn1—O1—S1	−40.93 (10)	O2—S1—C2—C3	82.66 (17)
O4^i^—Zn1—O1—S1	139.07 (10)	O1—S1—C2—C3	−36.38 (18)
O1W^i^—Zn1—O1—S1	49.40 (10)	Zn1—O4—C3—C2	6.9 (3)
O1W—Zn1—O1—S1	−130.60 (10)	Zn1—O4—C3—C4	−173.69 (14)
O1^i^—Zn1—O4—C3	−173.83 (17)	C1—C2—C3—O4	177.73 (19)
O1—Zn1—O4—C3	6.17 (17)	S1—C2—C3—O4	6.0 (3)
O1W^i^—Zn1—O4—C3	−85.06 (18)	C1—C2—C3—C4	−1.7 (3)
O1W—Zn1—O4—C3	94.94 (18)	S1—C2—C3—C4	−173.44 (15)
C5—N1—C1—C2	0.0 (3)	O4—C3—C4—C5	−177.7 (2)
N1—C1—C2—C3	0.9 (3)	C2—C3—C4—C5	1.8 (3)
N1—C1—C2—S1	173.18 (17)	C3—C4—C5—N1	−1.0 (4)
O3—S1—C2—C1	32.14 (19)	C1—N1—C5—C4	0.0 (3)

Symmetry codes: (i) −*x*+1, −*y*+1, −*z*+1.

### Hydrogen-bond geometry (Å, °)

**Table:**

*D*—H···*A*	*D*—H	H···*A*	*D*···*A*	*D*—H···*A*
O1w—H1···O2^i^	0.84 (1)	2.05 (2)	2.797 (2)	147 (2)
O1w—H2···O4^ii^	0.84 (1)	1.92 (1)	2.744 (2)	171 (3)
N1—H3···O3^iii^	0.85 (1)	1.91 (1)	2.754 (2)	175 (3)

Symmetry codes: (i) −*x*+1, −*y*+1, −*z*+1; (ii) *x*+1, *y*, *z*; (iii) *x*−1, −*y*+1/2, *z*−1/2.
